# *Anomoneura taiwanica* sp. nov. (Hemiptera, Psylloidea, Psyllidae), a new jumping plant-louse species from Taiwan associated with *Morus australis* (Moraceae)

**DOI:** 10.3897/zookeys.917.36727

**Published:** 2020-03-09

**Authors:** Geonho Cho, Yi-Chang Liao, Seunghwan Lee, Man-Miao Yang

**Affiliations:** 1 Insect Biosystematics Laboratory, Research Institute of Agriculture and Life Science, Department of Agricultural Biotechnology, Seoul National University, 151-921, South Korea Seoul National University Seoul South Korea; 2 Department of Entomology, National Chung Hsing University, 145, Xinda Rd., Taichung 402, Taiwan National Chung Hsing University Taichung Taiwan

**Keywords:** Asia, DNA barcoding, mulberry, new species, Oriental region, psyllid, Sternorrhyncha, taxonomy

## Abstract

*Anomoneura taiwanica***sp. nov.** (Hemiptera, Psylloidea, Psyllidae, Psyllinae) is described based on samples from Taiwan that were previously misidentified as *A. mori* Schwarz, 1896. Morphological and genetic differences between the two species, as well as their distribution, are detailed and discussed. Comments on the pest status of *Anomoneura* spp. in East Asia are also provided.

## Introduction

Psyllids (Hemiptera, Psylloidea) are small phytophagous insects, ranging from 1−10 mm. About 4,000 species are known worldwide (Burckhardt and Ouvrard 2012). Some species are important pests of crops and forest trees, damaging plants by direct feeding and vectoring plant diseases. Psyllids are generally host specific and related psyllid species often develop on related host taxa (Ouvrard et al. 2015).

Knowledge of the psyllid fauna of Taiwan was first developed by foreign researchers during the first half of the 20^th^ century (Kuwayama 1908, 1910, 1931; Enderlein 1914). More comprehensive taxonomic work was later carried out by C.T. Yang and others (Yang 1984; Fang and Yang 1986; Yang et al. 1986; Lauterer et al. 1988; Fang 1990; Yang et al. 2004, 2009, 2013; Liao et al. 2016; Liao and Yang 2018). In total, nearly 150 species from 46 genera with representatives of all eight currently recognized families of Psylloidea have been recorded in Taiwan.

Until now, *Anomoneura* Schwarz, 1896 was considered a monotypic genus of jumping plant-lice (Hemiptera, Sternorrhyncha, Psylloidea, Psyllidae, Psyllinae) and was only known from East Asia (Uhler 1896; Kwon 1983; Labina 2006; Li 2011; Liao and Yang 2018; Ouvrard 2019). The single species of the genus, *Anomoneura mori* Schwarz, 1896, is a serious pest of mulberry (*Morus* spp.; Moraceae) (Kuwayama 1971). The species causes damage to mulberry plants by excessive removal of phloem sap and soiling fruit by secreting a large amount of honeydew and thread-like wax masses. Very recently *A. mori* was reported from Taiwan (Liao and Yang 2018). We have recently come to the conclusion that the material from *Morus australis* from Taiwan was misidentified by Liao and Yang (2018) and is actually an undescribed *Anomoneura* species that is morphologically similar to *A. mori*. We formally describe the new species here.

## Material and methods

Material for this study was examined from the following institutions: Korea National Arboretum, Pocheon, Korea (**KNA**); National Chung Hsing University, Taichung, Taiwan (**NCHU**); Naturhistorisches Museum, Basel, Switzerland (**NHMB**); National Institute of Biological Resources, Incheon, Korea (**NIBR**); National Museum of Natural Science, Taichung, Taiwan (**NMNS**); National Pingtung University of Science and Technology, Pingtung, Taiwan (**NPUST**); National Taiwan University, Taipei, Taiwan (**NTU**); Seoul National University, Seoul, Korea (**SNU**); Taiwan Agriculture Research Institute, Taichung, Taiwan (**TARI**); and Zoological Institute, Russian Academy of Sciences, St Petersburg, Russia (**ZIN**).

Morphological terminology follows mostly Ossiannilsson (1992), Hollis (2004), and Yang et al. (2009). For molecular diagnosis, the COI-tRNA^leu^-COII fragment of mitochondrial DNA was used, as it is usually effective for comparison of closely related psyllid species (Cho et al. 2020). Protocols for DNA extraction, amplification, sequencing, sequence alignment, and phylogenetic analysis were followed from Cho et al. (2020). In addition to the material of *Anomoneura*, two *Acizzia* species (Psyllidae, Acizziinae) were included in the phylogenetic analysis as outgroups (Table 1). K2P distance and *p*-distance were computed using MEGA 6 (Tamura et al. 2013). Nomenclature for genetic sequences in Table 1 follows Chakrabarty et al. (2013).

**Table 1.** *Anomoneura* and *Acizzia* sequences of COI-tRNA^leu^-COII used in this study.

Species	Specimen Catalog #	Country	GenBank #	GenSeq
*Anomoneura mori*	SNU 4-1	South Korea	MN879300	genseq-4
	SNU 4-2	South Korea	MN879301	genseq-4
	SNU 161-1	Japan	MN879307	genseq-4
	SNU 161-2	Japan	MN879308	genseq-4
	SNU 161-3	Japan	MN879306	genseq-4
	SNU 161-4	Japan	MN879309	genseq-4
	SNU 161-6	Japan	MN879310	genseq-4
*Anomoneura taiwanica*	SNU 159-1	Taiwan	MN879302	genseq-4
	SNU 159-2	Taiwan	MN879305	genseq-4
	SNU 159-3	Taiwan	MN879303	genseq-4
	SNU 159-4	Taiwan	MN879304	genseq-4
*Acizzia jamatonica*	SNU 1-2	South Korea	MK039677	genseq-4
*Acizzia sasakii*	SNU 2-2	South Korea	MK039678	genseq-4

## Taxonomy

### Key to adults of *Anomoneura* species

**Table:** 

1	Forewing with obliquely truncate apex, membrane with scattered dark dots (Fig. 1). Paramere, in profile, slightly broader, clavate with apical tooth directed upwards and slightly forwards (Figs 3, 5). Apical dilation of distal segment of aedeagus, in profile, narrowly oblong (Fig. 7). Female proctiger with dorsal margin, in profile, slightly sinuate posterior to circumanal ring; circumanal ring as long as one third of proctiger length (Fig. 9)	***A. mori* Schwarz**
–	Forewing with nearly rounded apex, membrane with blurred dark patches (Fig. 2). Paramere, in profile, slightly narrower, lanceolate with apical tooth curved towards the rear (Figs 4, 6). Apical dilation of distal segment of aedeagus, in profile, broader, irregularly spherical (Fig. 8). Female proctiger with dorsal margin, in profile, nearly straight posterior to circumanal ring; circumanal ring slightly shorter than half of proctiger length (Fig. 10)	***A. taiwanica* sp. nov.**

#### Anomoneura taiwanica

Taxon classificationAnimaliaHemipteraPsylloidea

Cho & Liao sp. nov.

F101D790-7070-527A-9A82-13DCAA18C423

http://zoobank.org/D1DBFB0B-BDC7-4FB4-8990-980F7055893D

Figs 2 , 4 , 6 , 8 , 10

Anomoneura mori sensu Liao and Yang 2018: 604: figs 2–5; nec Schwarz in Uhler 1896: 296.

##### Type locality.

Taiwan, Miaoli County, Nanzhuang, Daping, 24°32'07"N, 120°58'11"E, 525 m alt.

##### Type material.

***Holotype***: Taiwan • ♂; Miaoli Co., Nanzhuang, Daping; 24°32'07"N, 120°58'11"E; 525 m a.s.l.; 29 Apr. 2011; Y.C. Liao leg.; *Morus australis*; NCHU, dry mounted. ***Paratypes***: Taiwan: • 108 ♂, 103 ♀, 6 immatures; same data as for holotype; NCHU, NMNS, NHMB, dry and slide mounted • 7 ♂, 12 ♀; Miaoli Co., Dongho; 24°32'12"N, 121°01'30"E; 1040 m a.s.l.; 19 Apr. 2012; Y.C. Liao leg.; *M. australis*; NCHU, dry mounted • 1 ♀; Nantou Co., Huisun forest station; 24°05'23"N, 121°01'50"E; 694 m a.s.l.; 20 Apr. 2011; Y.C. Liao leg.; *M. australis*; NCHU, dry mounted • 10 ♂, 3 ♀, 14 immatures; same locality as for preceding; 28 Mar. 2011; T.J. Hsieh leg.; *M. australis*; NCHU, dry and slide mounted.

##### Other material examined

(not included in the type series). Taiwan: • 39 ♂, 17 ♀; same data as holotype; NCHU, SNU, in 70% and 99% ethanol • 12 ♂, 11 ♀, 15 immatures; Taoyuan City, Fuxing, Xiaowulai; 24°47'37"N, 121°23'07"E; 563 m a.s.l.; 23 Apr. 2018; Y.C. Liao leg.; *M. australis*; NCHU, SNU, in 70% and 99% ethanol • 16 ♂, 17 ♀, 59 immatures; Taoyuan City, Fuxing, Shihmen reservoir; 24°49'19"N, 121°14'23"E; 228 m a.s.l.; 21 Apr. 2018; Y.C. Liao leg.; *M. australis*; NCHU, in 70% ethanol • 1 ♀; Hsinchu Co., Chienhsi; 5 Nov. 1981, K.S. Lin leg.; TARI, dry mounted • 1 ♂; Hsinchu Co., Chutung; 5 Apr. 1981; T.C. Hsu leg.; NTU, dry mounted • 1 ♂; Taichung City, Dongshi; 1 Feb. 2002; W.H. Chen leg.; NPUST, dry mounted • 19 ♂, 22 ♀; Nantou Co., Wushe; 15 Apr. 1987; L.J. Tang leg.; TARI, dry mounted • 1 ♂, 1 ♀; Nantou Co., Tungpu; 28 Apr.–2 May 1981; T. Lin and C.J. Lee leg.; TARI, dry mounted.

##### Diagnosis.

Forewing oblong-oval with unevenly rounded apex, membrane with dark patches fused and blurred in apical two thirds (Fig. 2). Paramere, in profile, lanceolate, tapering to apex, with a subacute apical tooth weakly curved towards the rear (Figs 4, 6). Distal segment of aedeagus sinuous, nearly the same thickness in basal three quarters, apical dilation, in profile, forming irregular sphere (Fig. 8). Female proctiger with dorsal margin, in profile, nearly straight posterior to circumanal ring, which is slightly shorter than half of proctiger length (Fig. 10).

**Figures 1–10.**
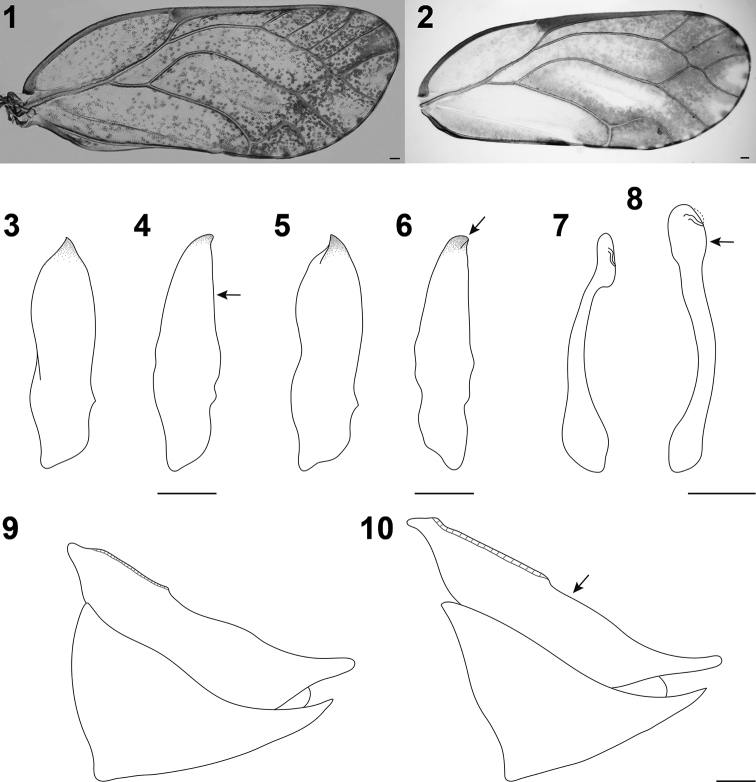
Diagnostic characters of *Anomoneura* spp. **1, 3, 5, 7, 9***A. mori* Schwarz (specimens from Korea) **2, 4, 6, 8, 10***A. taiwanica***sp. nov.** (specimens from Taiwan): **1, 2** forewing **3, 4** paramere, inner surface **5, 6** paramere, outer surface **7, 8** distal segment of aedeagus **9, 10** female terminalia. Scale bars: 0.1 mm.

##### Description.

A complete description including measurements and illustrations of both sexes and the fifth instar immature were given by Liao and Yang (2018).

##### Etymology.

The new species name is derived from the country where the type material was collected, Taiwan, and the Latin suffix -*icus*, -*a*, -*um* (belonging to). Adjective.

##### Distribution.

Taiwan (Liao and Yang 2018).

##### Host plant.

*Morus australis* Poir. (Moraceae), confirmed by the presence of immatures (Liao and Yang 2018).

#### Anomoneura mori

Taxon classificationAnimaliaHemipteraPsylloidea

Schwarz, 1896

CF428CAE-7B29-55CD-B4F5-9D2552E52CC5

Figs 1 , 3 , 5 , 7 , 9

Anomoneura mori Schwarz in Uhler, 1896: 296; Kwon 1983: 28; Li 2011: 586.
Anomoneura koreana Klimaszewski, 1963: 92; synonymised by Kwon 1983: 28.

##### Material examined.

China: • 1 ♂; Sichuan, Wliang-Zhengzhou; 18 Sep. 1993; Pomanin leg.; ZIN, dry mounted. Russia: • 11 ♂, 10 ♀; Kunashir Island, Tretyakovo; 8 Aug. 1971; Ermolenko leg.; ZIN, dry and slide mounted • 6 ♂, 10 ♀; same locality as for preceding; 16 Jun. 1973; Kerzhner leg.; ZIN, dry mounted. Japan: • 1 ♂, 1 ♀; Shikoku; 3 Jun. 1953; K. Sasaki leg.; ZIN, dry mounted • 2 ♂, 2 ♀; Kyushu, Mt. Homan, Chikuzen; 12 Jun. 1962; *Morus bombycis*; Y. Miyatake leg.; ZIN, dry mounted • 4 ♂, 4 ♀; Honshu, Ibaraki Pref., Tsukuba City; Fujimoto leg.; 30 May 2003; *Morus* sp.; H. Inoue leg.; SNU, in 95% ethanol • 2 ♂, 2 ♀; same locality as for preceding; 4 Jun. 2004; *M. alba*; H. Inoue leg.; SNU, dry mounted • 1 ♂, 2 ♀, 3 immatures; Kyushu, Kumamoto Pref., Amakusa-shimoshima Is., Amakusa City, Ushibuka, Mogushi; 32°211'N, 130°005'E; 5 m a.s.l.; 25 May 2015; *Morus* sp.; H. Inoue leg.; SNU, dry mounted and in 95% ethanol • 5 ♂, 5 ♀; Kyushu, Nagasaki Pref., Tsushima Is., Tsushima City, Izuhara, Kamizaka; 380 m a.s.l.; 6 Jun. 2018; *Morus* sp.; H. Inoue leg.; SNU, dry mounted and in 95% ethanol. South Korea: • 2 ♂; Jeollabuk-do, Mt. Mayi; 11 May 1980; Y.J. Kwon leg.; NIBR, dry mounted • 2 ♂; Gangwon-do, Mt. Seolak; 29 May 1980; Y.J. Kwon leg.; NIBR, dry mounted • 1 ♂, 1 ♀; Gangwon-do, Mt. Obong; 17 May 1981; Y.J. Kwon leg.; NIBR, dry mounted • 1 ♂, 1 ♀; Gyeongsangbuk-do, Is. Ulleungdo; 26 May 1981; Y.J. Kwon leg.; NIBR, dry mounted • 1 ♂, 3 ♀; same locality as for preceding; 27 May 1981; Y.J. Kwon leg.; NIBR, dry mounted • 3 ♂; same locality as for preceding; 1 Oct. 1981; Y.J. Kwon leg.; NIBR, dry mounted • 1 ♂; same locality as for preceding; 3 Oct. 1981; Y.J. Kwon leg.; NIBR, dry mounted • 2 ♂, 3 ♀; Gyeongsangnam-do, Mt. Geumsan; 29 Mar. 1982; Y.J. Kwon leg.; NIBR, dry mounted • 1 ♀; Gyeonggi-do, Anyang-si; 19 Jun. 1992; S.J. Park leg.; SNU, dry mounted • 1 ♂, 1 ♀; Gyeongsangnam-do, Mt. Cheonhwang; 12 Sep. 1999; Y.J. Kwon leg.; NIBR, dry mounted • 1 ♂; Gyeonggi-do, Yangpyeong-gun, Yongmun-myeon, Sinjeom-ri, Mt. Yongmun; 24 Jun. 2009; S.H. Lee leg.; SNU, dry mounted • 4 ♂, 1 ♀; Gyeongsangnam-do, Miryang-si, Danjang-myeon, Mt. Jaeyak; 30 Jun. 2011; S.W. Cheong leg.; NIBR, dry mounted • 1 ♂; Gangwon-do, Inje-gun, Buk-myeon, Yongdae-ri, Yongdae National Recreation Center; 19 Jun. 2013; G. Cho leg.; SNU, slide-mounted • 4 ♂, 1 ♀; Jeollanam-do, Gwangyang-si, Ongnyong-myeon, Chusan-ri, Mt. Baegun; 24 Aug. 2013; G. Cho leg.; SNU, dry and slide-mounted • 5 ♂, 2 ♀, 51 immatures; Gyeonggi-do, Seongnam-si, Bundang-gu, Munjeong-ro 151; 29 May 2014; *M. alba*; G. Cho leg.; KNA, SNU, dry mounted, in 95% ethanol.

## Discussion and conclusion

*Anomoneura mori* was described from Japan by Schwarz in Uhler (1896) and subsequently reported from the Korean Peninsula (Chon 1963; Klimaszewski 1963), the Russian Far East (Gegechkori and Loginova 1990; Labina 2006), China (Li 2011), and most recently from Taiwan (Liao and Yang 2018). The Taiwanese population of *Anomoneura* showed some morphological differences from *A. mori* specimens from other countries, but unfortunately, this was overlooked (Liao and Yang 2018).

**Figure 11.**
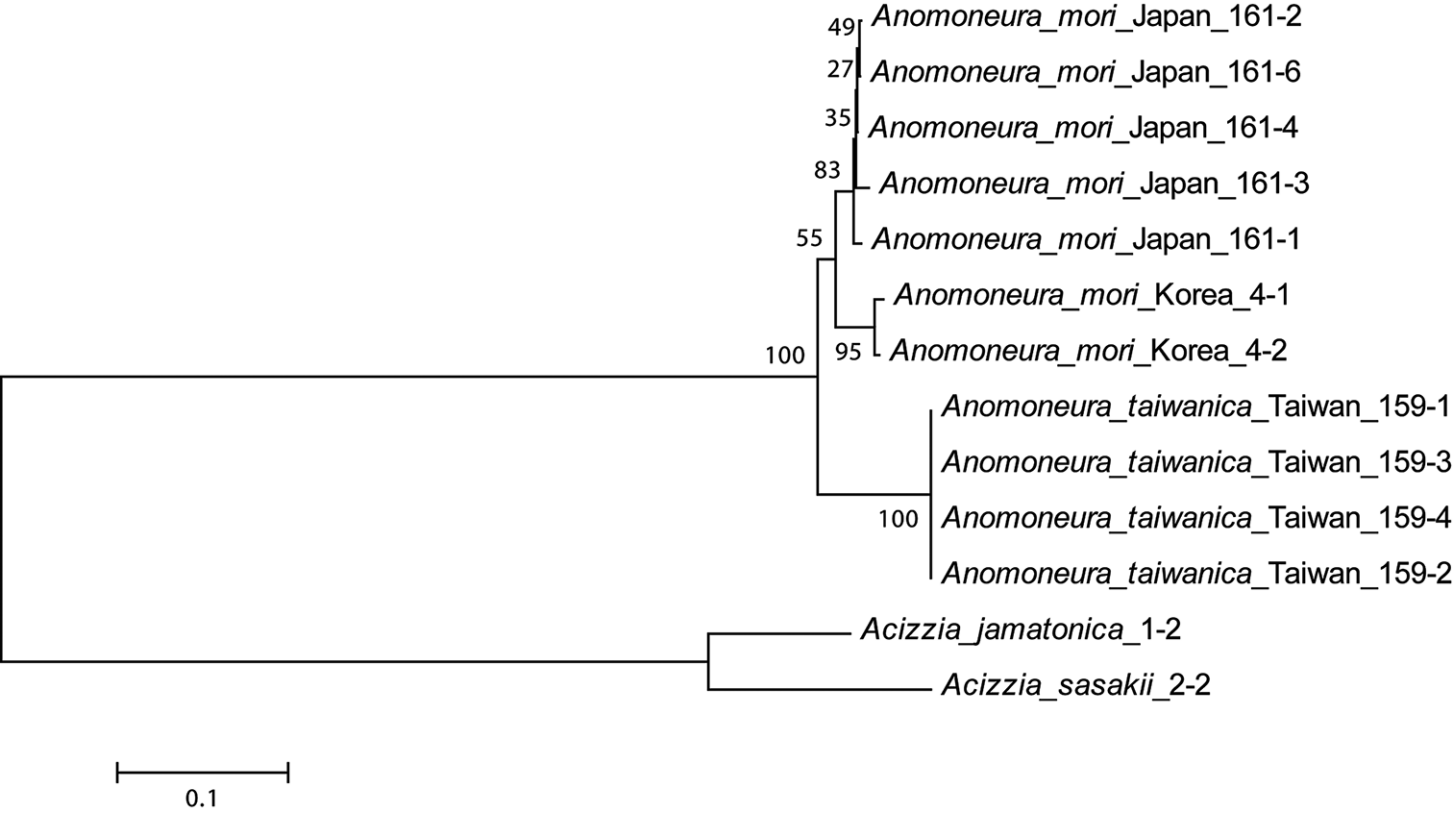
A neighbor-joining tree based on the Kimura 2-parameter genetic distance between COI-tRNA^leu^-COII sequences. Bootstrap support values are shown at the branch points and are based on 1,000 replications.

*Anomoneura taiwanica* sp. nov. resembles *A. mori* in the structure of the head, the general structure of the forewing, and a similar host association with plants of the genus *Morus*. *Anomoneura taiwanica* sp. nov. differs from *A. mori* in the details of the forewing, paramere, distal segment of aedeagus, and female proctiger (see the key above, Table 2, and Figs 1–10). The shape of the vein Cu_1a_ of the forewing is quite variable, curved at 90° to 100° in examined *Anomoneura* material, including Taiwanese populations, which likely reflects an intraspecific variation in both species (Figs 1, 2). No significant morphological differences were found between immatures of these taxa. We analysed DNA sequence fragments of *A. mori* from Japan and Korea and *A. taiwanica* sp. nov. from Taiwan. Sequences from Japan and Korea showed no significant genetic divergence from each other (1.8% *p*-distance and K2P distance). However, the difference of those populations from *A. taiwanica* sp. nov. was significantly higher (9.0% p-distance, 9.7% K2P distance) (Fig. 11). A 3% genetic distance has been considered supportive for diagnosis of taxa at the species level for psyllids in general (Percy 2003; Martoni et al. 2018).

**Table 2.** Differences between *Anomoneura mori* and *Anomoneura taiwanica* sp. nov.

Character	*A. mori*	*A. taiwanica* sp. nov.
Forewing apex	obliquely truncate	rounded
Forewing maculation	partly fused in apical part	fused and blurred
Paramere	clavate	lanceolate
Distal segment of aedeagus	curved posteriad, narrowing toward apex	sinuous, nearly as thick basally as apically
Dilation of distal segment of aedeagus	narrowly oblong	irregularly spherical
Dorsal margin of female proctiger	sinuate	nearly straight
Distribution	China, Japan, Korea, Russia	Taiwan
Host plant	*Morus alba*, *M. australis*	*M. australis*

Mulberry (*Morus* spp.) is important to sericulture as it is also the host plant for silkworms. Due to this, damage caused by *Anomoneura* can significantly affect the silkworm industry in both Taiwan and abroad. Populations of *A. mori* can remove large quantities of plant sap, produce masses of wax threads, and secrete a large amount of honeydew. This causes negative effects on plant growth and diminishes mulberry leaves which devalues the silkworms cocoons (Kuwayama 1971; Kim and Park 2016). A recent field survey by Liao and Yang (2018) in Taiwan showed that *A. taiwanica* has the same life history as *A. mori*, which indicates that both species are serious pests of mulberry trees. However, at present, *A. taiwanica* may not cause serious economic loss in Taiwan due to a decline of the silk industry in the late 1990s. Just a few silkworm farmers remain in Taiwan, and these are merely for traditional or educational purposes (Jiang 2007).

Liao and Yang (2018) considered *A. taiwanica* sp. nov. (which they had misidentified as *A. mori* at the time) as an exotic invader in Taiwan because earlier surveys of *Morus* trees did not identify this species as present in the country (Yang 1984; Yang et al. 1986). Furthermore, *A. mori* is widely distributed in the temperate climatic zone of East Asia at higher latitudes than *A. taiwanica*, which is known only from the subtropical climate of northern Taiwan. The recent increase in the import of various plant seedlings from China to Taiwan has often accelerated the establishment of exotic species, such as *Cacopsylla chinensis* (Yang et al. 2004). Given this fact, *A. taiwanica* may have originated in southern China. To confirm this hypothesis, more material of *Anomoneura* from China should be examined to reveal a potentially wider distribution of *A. taiwanica*.

## Supplementary Material

XML Treatment for Anomoneura taiwanica

XML Treatment for Anomoneura mori
